# Optimizing Patient Selection for Irreversible Electroporation of Locally Advanced Pancreatic Cancer: Analyses of Survival

**DOI:** 10.3389/fonc.2021.817220

**Published:** 2022-01-13

**Authors:** Matthew R. Woeste, Khaleel D. Wilson, Edward J. Kruse, Matthew J. Weiss, John D. Christein, Rebekah R. White, Robert C. G. Martin

**Affiliations:** ^1^ Division of Surgical Oncology, Department of Surgery, University of Louisville School of Medicine, Louisville, KY, United States; ^2^ Department of Surgery, Section of Surgical Oncology, Augusta University Medical Center, Augusta, GA, United States; ^3^ Department of Surgery, Division of Surgical Oncology, Johns Hopkins University, Baltimore, MD, United States; ^4^ Department of Surgery, Division of Gastrointestinal Surgery, University of Alabama, Birmingham, AL, United States; ^5^ Gastrointestinal Cancer Unit, University of California San Diego Moores Cancer Center, San Diego, CA, United States

**Keywords:** locally advanced pancreatic cancer, irreversible electroporation (IRE), overall survival, patient selection, recurrence, progression free survival

## Abstract

**Background:**

Irreversible electroporation (IRE) has emerged as a viable consolidative therapy after induction chemotherapy, in which this combination has improved overall survival of locally advanced pancreatic cancer (LAPC). Optimal timing and patient selection for irreversible electroporation remains a clinically unmet need. The aim of this study was to investigate preoperative factors that may assist in predicting progression-free and overall survival following IRE.

**Methods:**

A multi-institutional, prospectively maintained database was reviewed for patients with LAPC treated with induction chemotherapy followed by open-technique irreversible electroporation from 7/2015-5/2019. RECIST 1.1 criteria were used to assess tumor response and radiological progression. Overall survival (OS) and progression-free survival (PFS) were recorded. Survival analyses were performed using Kaplan Meier and Cox multivariable regression analyses.

**Results:**

187 LAPC patients (median age 62 years range, 21 – 91, 65% men, 35% women) were treated with IRE. Median PFS was 21.7 months and median OS from diagnosis was 25.5 months. On multivariable analysis, age ≤ 61 (HR 0.41, 95%CI 0.21-0.78, p<0.008) and no prior radiation (HR 0.49, 95%CI 0.26-0.94, p=0.03) were positive predictors of OS after IRE. Age ≤ 61(HR 0.53, 95%CI, 0.28-.99, p=0.046) and FOLFIRINOX followed by gemcitabine/abraxane induction chemotherapy (HR 0.37,95%CI 0.15-0.89, p=0.027) predicted prolonged PFS after IRE. Abnormal CA19-9 values at the time of surgery negatively impacted both OS (HR 2.46, 95%CI 1.28-4.72, p<0.007) and PFS (HR 2.192, 95%CI 1.143-4.201, p=0.018) following IRE.

**Conclusions:**

Age, CA 19-9 response, avoidance of pre-IRE radiation, and FOLFIRINOX plus gemcitabine/abraxane induction chemotherapy are prominent factors to consider when referring or selecting LAPC patients to undergo IRE.

## Introduction

The diagnosis of pancreatic ductal adenocarcinoma (PDAC) continues to have a challenging prognosis, but improvements in multi-disciplinary care have raised the overall survival rates to 10% for all stages (1). In 2020, an estimated 47,050 patients will die of this disease representing the third highest cancer causing mortality rate (2). Modern systemic chemotherapy followed by surgical resection has dramatically improved the standard of care, however this is only available to approximately 10-20% of the patients diagnosed each year. Forty percent of patients present with local invasion [stage III - locally advanced pancreatic cancer (LAPC)] and are most often prescribed poorly responsive systemic palliative chemotherapy (3). Multimodality induction chemotherapy with folinic acid, 5-fluorouracil, leucovorin, irinotecan, and oxaliplatin (FOLFIRINOX) has prolonged overall survival (OS) to 12 months, however continued response rates after 4-6 months are poor due to the cumulative toxicity, need for dose delay, dose reduction, or complete termination of this active treatment (4–6). A current clinical unmet need is to develop and offer clinically effective therapies to consolidate the response of systemic chemotherapy in patients with unresectable LAPC after 3-4 months of induction therapy.

Historically, LAPC is deemed unresectable to conventional surgical intervention and is thought of as a continuum of metastatic disease. Yet additional consolidative treatment options for LAPC following induction chemotherapy exist and have been successfully utilized with improved outcomes. Irreversible electroporation (IRE), a non-thermal ablation technology, has begun to gain acceptance within the last decade (7–9) IRE induces cellular apoptosis without disrupting surrounding tissue structural integrity (10). Martin et al. demonstrated IRE is a safe and effective treatment of LAPC with initial improvements in median OS to 25.3 months (11). These results were confirmed with combination of chemotherapy and IRE improving median overall survival to 30.7 months, critically implicating IRE to be included in the multimodal treatment of LAPC (12).

The aim of this study was to evaluate LAPC pre-procedural/preoperative patient predictors of progression-free survival (PFS) and OS following induction chemotherapy to better guide patient selection for IRE utilization as part of a multimodal treatment for LAPC.

## Methods

### Participants

An Institutional Review Board (IRB) approved single arm study of patients diagnosed National Comprehensive Cancer Network (NCCN) stage III LAPC of patients treated by IRE between July 2015 and May 2020 was evaluated. This prospective pancreatic cancer registry represents a multi-institutional collection of patients with radiographic stage III LAPC all of whom were treated with IRE (13). Six participating institutions included the University of Louisville, University of South Florida, Augusta University, University of Alabama, and University of California, San Diego. The registry is open to any center worldwide that wishes to participate and collaborate with their data (12). All patients provided written informed consent. A diagnosis of LAPC disease was established by biopsy proven adenocarcinoma of the pancreas with unreconstructable venous involvement or greater than 180° encasement of their superior mesenteric artery (SMA) or celiac artery without evidence of metastatic lesions (12, 14, 15). Patients were also further sub-classified by our recent Stage III classification sub-types (16). Patients were further considered for inclusion in the study if the treating physician at the aforementioned participating institutions believed that ablation of their soft tissue would be feasible in the care of their disease, as has been previously described and outlined (17–19). Staging included triple phase computed tomographic (CT) scan with less than 1.5-mm cuts at the time of diagnosis and repeated 1-2 weeks prior to IRE (11, 20). To aide in post ablation follow up and response, positron emission tomography (PET-CT) scanning was initiated in January of 2019.

Inclusion criteria involved eligible patients underwent induction therapy consisting of chemotherapy and/or external beam radiation therapy following each respective institution’s protocol. Patients underwent restaging evaluation 4 to 6 weeks after induction therapy *via* repeat triple-phase CT scan and serum tumor markers. Those with evidence of disease progression were excluded. Patients found on restaging to be free of metastatic disease and without primary tumor progression were included and received either IRE *in situ* or IRE with resection. All patients included were Stage III LAPC based on pre-operative imaging. Patient selection is critical to the safety and efficacy of IRE for LAPC. This has been outlined extensively in previous publications (17, 19, 21).

Key exclusion criteria were patients with implanted cardiac pacemaker or defibrillators unable to be deactivated, non-removable implants with metal parts within 1 cm of the target lesion, a myocardial infarction within 3 months, or unsuitable for general endotracheal anesthesia. All presented data was collected and maintained in a prospective manner. Adverse events were summarized using the National Cancer Institute (NCI) Common Terminology Criteria Adverse Event (CTCAE), version 3.0 and graded *via* Clavien-Dindo classification (22).

### Interventions

#### Systemic Therapy

FOLFIRINOX based chemotherapy was administered for at least 6 to 8 cycles on a 14-day cycle, commonly using standard dosing per standard of care and each institutions management (23). Similarly Gemcitabine and abraxane were administered using standard dosing per standard of care and each institutions management on days 1, 8, and 15 every 4 weeks (24). Patients were restaged after induction chemotherapy *via* repeat triple-phase CT scan and serum tumor markers and evaluated by a multidisciplinary team. All patients with evidence of disease progression were excluded. Only patients that had received FOLFIRINOX, gemcitabine and abraxane, or single agent gemcitabine as an induction therapy prior to IRE were included in survival analyses.

### Irreversible Electroporation

Patients found to be free of metastatic disease and without primary tumor progression on re-staging were included and further received an open surgical *in situ* IRE based on intra-operative findings and location of the primary tumor as described previously (11, 25). Open Insitu-IRE was performed utilizing AngioDynamics NanoKnife system, as previously described and were performed by surgeons in the operating room (17, 19, 26). All participating institutions utilized the registry protocol for standardization of settings setup and delivery of energy during the IRE procedure as previously reported (12, 17, 19, 27).

### Post-Procedure Evaluation and Follow Up

After IRE follow-up imaging *via* triple-phase CT scan was performed during the immediate postoperative period to evaluate for early complications, assess the patency of vital structures, and to establish a baseline of the post-ablation bed, as has been previously reported (12, 28, 29). Ablation success was evaluated at 3 months post-IRE treatment *via* triple-phase CT scan following pancreatic imaging protocol, along with CA19-9, and PET-CT. Ablation success and recurrence have been previously defined (15). Participating institutions standardized utilization of CT scans to avoid the difficulty encountered with cross-comparing CT scans to MRI or CT scan to PET scans in previous studies. Response and progression were evaluated using the international criteria proposed by RECIST 1.1 (21). Serial imaging over at least two months were subsequently used to detect recurrence through study comparison in combination with clinical and serum CA19-9 studies. If equivocal findings where seen on CT then a PET was obtained to either confirm or refute local and/or regional recurrence when required.

### Statistical Analyses

OS was defined as the time from the start of treatment to the date of death, due to any reason. PFS was defined as the time from the start of initial IRE treatment to the date of first observed disease progression. The rates of OS and PFS were estimated by Kaplan-Meier method. Multivariable Cox survival regression was performed to determine independent predictors of PFS and OS after backward selection (criterion p<0.05) to include all variables of interest. All statistical analyses were performed using SAS version 9.3 (SAS Institute, Cary, NC), and p values less than 0.05 were considered significant.

## Results

An intention to treat analysis of 187 patients who met inclusion criteria underwent IRE for stage III LAPC. Baseline demographics of the study cohort are represented in **Table 1**. Sixty five percent of the cohort was male with a median age of 62 years. The majority of the population were of White (55%) or Asian (39%) ethnicity. Preoperative tumor characteristics and chemotherapy and radiation interventions are represented in **Table 2**. Thirty eight percent of tumors were located in the head of the pancreas with 53% of patients had tumors > 3cm in greatest diameter. Preoperative radiation therapy was administered to 28% of the cohort. All patients in this study received induction preoperative chemotherapy. Forty-two patients (22%) within the cohort received FOLFIRINOX alone, 62 (33%) had FOLFIRINOX + gemcitabine and abraxane, and 19 (10%) were administered gemcitabine alone, respectively. A majority of patients (90%) had abnormal CA19- levels at the time of diagnosis once their bilirubin’s were normalized.

**Table 1:** Patient characteristics at baseline of entire cohort.

Characteristic	Study cohort (n=187)
Age (years), median, (IQR)	62 (21 - 91)
Male gender, n (%)	121 (65)
BMI, median (IQR)	25.7 (14 - 41)
**Ethnicity, n (%)**	
Asian	73 (39)
Black/African American	8 (4)
Native Hawaiian or other Pacific Islander	1 (0.5)
White	102 (55)
Unknown/not reported	2 (1)
Other	1 (0.5)
**Past medical history, n (%)**	
Cardiac	16 (9)
Diabetes	25 (13)
Hypertension	32 (17)
Liver dysfunction	2 (1)
Pancreatitis	12 (6)
Pulmonary	7 (4)
Vascular	4 (2)
Tobacco History	23 (12)
Alcohol Abuse	8 (4)
**Past surgical history, n (%)**	
Appendectomy	13 (7)
Bile Stents	25 (13)
Cholecystectomy	30 (16)
Colon	4 (2)
Distal Pancreatectomy	4 (2)
Gastric Bypass	1 (0.5)
Orthopedic	13 (7)
TAH	10 (5)
Whipple	4 (2)
**Karnofsky Performance Score, n (%)**	
100%	77 (41)
90%	58 (10)
80%	47 (4)
70%	3 (2)
0%	2 (1)

IQR, interquartile range; BMI, body mass index; TAH, total abdominal hysterectomy.

**Table 2:** Tumor characteristics and neoadjuvant interventions.

Characteristic	Study cohort (n=187)
**Tumor location, n (%)**	
* Head*	71 (38)
* Head/body/neck*	76 (41)
* Body/neck/tail*	1 (0.5)
* Body/neck*	38 (20)
* Head/tail*	1 (0.5)
**Tumor size (cm), n (%)**	
* < 2*	4 (2)
* 2.1 - 3*	84 (45)
* >3*	99 (53)
**Vascular involvement, n (%)**	
* Single Arterial alone (SMA or Celiac)*	19 (10)
* Venous Alone*	19 (10)
* Arterial + Venous*	77 (41)
* Both Arterial (SMA and Celiac)*	72 (39)
**Induction chemotherapy, n (%)**	
FOLFIRINOX alone	42 (22)
FOLFIRINOX + gemcitabine/abraxane	62 (33)
Gemcitabine/abraxane	19 (10)
Other Combinations (5FU Alone, PARP Inhibitor, Gemcitabine and Cisplatin, FOLFOX, FOLFIRI)	64 (34)
**Prior radiation therapy, n (%)**	
* 3-D conformal*	37 (20)
* SBRT*	15 (8)
**Prior local therapy/surgery, n (%)**	
* IRE*	4 (2)
* Pancreatic resection*	3 (2)
**Percent drop in CA19-9, n (%)**	
* < 0*	32 (17)
* 0 - 58*	40 (21)
* 59 - 86*	34 (18)
* 87 - 96*	47 (25)
* > 96%*	34 (18)

SBRT, stereotactic body radiation therapy; IRE, irreversible electroporation; CA19-9, cancer antigen 19-9; FOLFIRINOX, folinic acid, 5-fluorouracil, irinotecan, and oxaliplatin.

**Table 3** outlines the operative characteristics, adjunctive procedures, and outcome measures for the cohort. The median time from diagnosis to IRE treatment was 4 months, with 56% of the cohort receiving additional adjunctive procedures at the time of IRE. Cholecystectomy (30%) and jejunostomy tube placement (45%) were the most common adjunctive procedures. Thirty-two patients (17%) had local recurrences and 49 (26%) experienced distant recurrence. Mean time to local recurrence was 16.4 months and 15.9 months to distant recurrence from IRE. The liver represented the most common location of distant progression (26%, 13/49). Adverse events following IRE occurred in 25% of the cohort and are presented in **Supplementary Table 1** stratified by CTCAE score.

**Table 3:** Operative characteristics, adjunctive procedures, and outcome measures.

Characteristic	Study cohort (n= 187)
Time from diagnosis to IRE treatment (mo.), median, (IQR)	4 (12 – 69)
Total IRE delivery time (min.), median, (IQR)	49 (2 - 307)
Total probe placement time (min.), median (IQR)	15 (2 - 21)
Total procedure time (min.) median, (IQR)	164 (50 - 540)
Time from procedure to discharge (days), median, (IQR)	7 (2 - 27)
Patients requiring pullback, n (%)	166 (89)
Patients with adjunctive procedures	105 (56)
**Adjunctive Procedure, n (%)**	
* Cholecystectomy*	57 (30)
* Distal pancreatectomy*	3 (2)
* Gastrojejunostomy*	39 (21)
* Hepaticojejunostomy*	27 (14)
* Jejunostomy-tube*	85 (45)
* Portal vein or SMV resection*	12 (6)
* Subtotal pancreatectomy with celiac resection*	5 (3)
* Whipple*	17 (9)
* Other(hernia repair, gastro-jejunostomy*	30 (16)
Patients receiving adjuvant therapy during follow-up, n (%)	74 (40)
Time to local recurrence from diagnosis, (mo.), mean, (IQR)	22.3 (0.1 - 77.1)
Time to local recurrence from IRE, (mo.), mean, (IQR)	16.4 (0 - 52.5)
Time to distant recurrence from diagnosis, (mo.), mean, (IQR)	21.9 (0.1 - 90.8)
Time to distant recurrence from IRE, (mo.), mean, (IQR)	15.9 (0 - 52.5)
PFS from diagnosis, (mo.), median, (IQR)	21.7 (0.1 – 77.1)
PFS from IRE, (mo.), median, (IQR)	16.1 (0 - 52.5)
OS from diagnosis, (mo.), median, (IQR)	25.5 (0.1 - 90.8)
OS from IRE, (mo.), median, (IQR)	22.4 (0 - 52.5)
**Recurrence type, n (%)**	
*Local*	32 (17)
*Distant*	49 (26)
**Location of distant progression, n (%)**	
*Bone*	1 (0.5)
*Liver*	25 (13)
*Lung*	13 (7)
*Ascites*	2 (1)
*Left suprarenal space*	1 (0.5)
*Omentum*	1 (0.5)
*Peritoneum*	4 (2)
*RUQ small intestine*	1 (0.5)
*Regional lymph node disease*	1 (0.5)
*Retroperitoneal lymph node*	1 (0.5)
*Abdominal wall*	1 (0.5)
*Around mesenteric artery*	1 (0.5)

IRE, irreversible electroporation; IQR, interquartile range; SMV, superior mesenteric vein; PFS, progression free survival; OS, overall survival; RUQ, right upper quadrant.

On univariate Kaplan Meier (KM) analyses, patients aged ≤ 61 experienced significantly longer OS (23.9 mo. vs 18.2 mo., 95% CI, 22 – 49.7, p=0.02) and PFS (22 mo. vs 12.5 mo., 95% CI, 18.8 – 30.4, p=0.04) after IRE therapy. Additionally, patients with ≤ 2 comorbidities demonstrated prolonged OS (21.6 mo. vs 12.4 mo., 95% CI 10.3 – 23.0, p=0.04) from IRE. Further, those specifically without diabetes compared to those with diabetes had increased OS (23.1 mo. vs 13.3 mo., 95% CI, 22 – 31.2, p=0.003) and PFS (20.6 mo. vs. 7.2 mo., 95% CI, 14.6 – 22.8, p=0.0003) following IRE.

Tumors sizes ≤ 3.6 cm were shown to have longer OS and PFS following IRE treatment (26.5 mo. vs. 18.2 mo., 95% CI, 22.5 – 52.5, p=0.0003), (22.8 mo. vs 0.4 mo., 95% CI, 19.4 – 35.9, p<0.0001). Vascular involvement was demonstrated in all of patients. When considering all levels of vascular involvement, those with ≤180° encasement demonstrated increased OS from initial diagnosis (52.8 mo. vs 22.7 mo., 95% CI, 7.7 – 90.8, p=0.006) and after IRE (52.5 mo. vs 2.4 mo., 95% CI, 4.8 – 52.5, p=0.01). PFS after initial diagnosis (52.8 mo. vs 6.8 mo., 95% CI, 17.5 – 77.1, p=0.001) and after receiving IRE (24.2 mo. vs 7.3 mo., p=0.005) was also significantly augmented by vascular involvements ≤180° (**Table 4**).

**Table 4:** Risk factors for overall and progression fee survival in stage III locally advanced pancreatic cancer patients.

Characteristic	KM Median (95% CI)	P value
**OS from diagnosis**		
CA19-9 change from diagnosis to IRE		**0.03**
Abnormal to normal	30.2 (22.5-43.5)	
Abnormal to abnormal	18.8 (15.4-22.7)	
Vascular involvement		
≤ 180°	52.8 (17.7-90.8)	**0.006**
> 180°	22.7 (18.4-24.7)	
Prior chemotherapy duration		
> 5 months	23.1 (17.4-34.7)	**0.04**
≤ 5 months	21.7 (15.7-24.4)	
**OS from IRE**		
Age		**0.02**
* ≤ 61*	23.9 (10-49.7)	
* > 61*	18.2 (13.3-23.1)	
CA19-9 change from diagnosis to IRE		**0.02**
Normal at IRE (≤ 37 U/mL)	17.5 (12.4-22.8)	
Abnormal at IRE (> 37 U/mL)	9.3 (5.9-13.3)	
Diabetes		**0.003**
Yes	13.3 (6.4-14.8)	
No	23.1 (22.0-31.2)	
Vascular involvement		**0.01**
≤ 180°	52.5 (4.8-52.5)	
> 180°	12.4 (6.6-16.4)	
Number of comorbidities		**0.04**
≤ 2	21.6 (10.3-23.9)	
> 2	12.4 (6.6-16.4)	
Prior chemotherapy		**0.01**
FOLFIRINOX + gemcitabine/abraxane	23.2 (19.4-35.9)	
FOLFIRINOX	13.3 (9.4-18.2)	
Gemcitabine/abraxane	12.4 (4.6-26.5)	
Prior radiation		**<0.0001**
Yes	12.3 (6.4-17.2)	
No	26.5 (22.7-6.3)	
Tumor size		**0.0003**
≤ 3.6	26.5 (22.5-2.5)	
> 3.6	18.2 (13.8-22.8)	
**PFS from diagnosis**		
Vascular involvement		**0.001**
≤ 180°	52.8 (17.5-77.1)	
> 180°	16.8 (15.7-18.4)	
Prior chemotherapy		**0.04**
FOLFIRINOX + Gemcitabine/abraxane	20.8 (17.5-24.7)	
FOLFIRINOX	18.7 (16.3-20.7)	
Gemcitabine/abraxane	15.5 (10.6-25.4)	
**PFS from IRE**		
Age		**0.046**
* ≤ 61*	22.0 (18.8-30.4)	
* > 61*	12.5 (8.9-186)	
CA19-9		
Normal at IRE	8.9 (7.0-11.5)	**0.006**
Abnormal at IRE	5.3 (3.7-6.6)	
Diabetes		**0.0003**
Yes	7.2 (4.3-13.8)	
No	20.6 (14.6-22.8)	
Vascular involvement		**0.005**
≤ 180°	24.2 (4.5-52.5)	
> 180°	7.3 (5.8-8.8)	
Prior chemotherapy		**<0.0001**
FOLFIRINOX + gemcitabine/abraxane	19.4 (14.5-23.2)	
FOLFIRINOX	8.7 (5.8-11.5)	
Gemcitabine/abraxane	8.8 (2.9-10.9)	
Preoperative radiation		**<0.0001**
Yes	6.7 (3.3-8.9)	
No	22.3 (18.9-29.3)	
Tumor size (cm)		**<0.0001**
≤ 3.6	22.8 (19.4-35.9)	
> 3.6	10.4 (8.2-6.1)	

OS, overall survival; KM, Kaplan Meier; CA19-9, cancer antigen 19-9; IRE, irreversible electroporation; PFS, progression free survival; FOLFIRINOX, folinic acid, 5-fluorouracil, irinotecan, and oxaliplatin. Bold values represent statistical significance with p values less than 0.05.

Chemotherapy duration of > 5 months significantly enhanced OS from initial diagnosis (23.1 mo. vs 21.7 mo., 95% CI, 17.4 – 34.7, p=0.04). Patients receiving FOLFIRINOX in conjunction with gemcitabine and abraxane compared to those receiving FOLFIRINOX alone, or gemcitabine/abraxane induction chemotherapy achieved greater PFS (19.4 mo. vs 8.7 mo. vs 8.8 mo., 95% CI, 14.5 – 23.2, p<0.0001) following IRE and from initial diagnosis (20.8 mo. vs 18.7 mo. vs 15.5 mo., 95% CI, 17.5 – 24.7, p=0.04). OS following IRE (23.2 mo. vs. 13.3 mo. vs. 12.4 mo., 95% CI 19.4 – 35.9, p=0.0013) was also significantly increased for those who received FOLFIRINOX with gemcitabine/abraxane induction chemotherapy. Prior radiation was a negative predictor of OS (26.5 vs. 12.3 mo., 95% CI, 22.7 – 46.3, p<0.0001) and PFS (22.3 mo. vs 6.7 mo., 95% CI, 18.9 – 29.3, p<0.0001) from IRE (**Table 4**).

Monitoring of response to neoadjuvant treatment by preoperative CA 19-9 levels was a significant factor in predicting survival of LAPC by IRE. OS from diagnosis and from IRE was significantly increased when patients with abnormally elevated CA19-9 levels reached normative values (<37U/mL) at the time of operation (30.2 mo. vs. 18.8 mo., 95% CI, 22.5 – 43.5, p=0.03), (17.5 mo. vs. 9.3 mo., 95% CI, 12.4 – 22.8, p =0.02). This finding was replicated on PFS measured from the time of surgery when CA19-9 reached normal levels at the time of IRE (8.9 mo. vs 5.3 mo., p=0.006) (**Table 1**).

On univariate Kaplan Meier (KM) analyses when the 25 patients who underwent pancreatectomy with IRE were compared two patients who underwent IRE alone there was a similar median overall progression free survival from diagnosis with margin accentuation 21.4 (11.1 two 24.3 parentheses versus insight 2 22.6 (19.9 two 25.4) months, p=0.0690, and a statistically significant improvement and overall progression free survival from IRE treatment pancreatectomy with IRE 10.2 (3.4 to 31.5) versus *in-situ* of 21.9 (16.1 to 23.3) months, p=0.0452 (**Supplemental Figure 1**). Overall survival from diagnosis for pancreatectomy with IRE was 24.9 (12.6 to 39.1) versus 29.4 (23.1 to 36.2) months, p=0.23 and from IRE treatment was 15.6 mon (7.6 to 33.6) vs IRE *in-situ* 23.2 (22 to 31.2) months, p=0.076.

**Figure 1:**
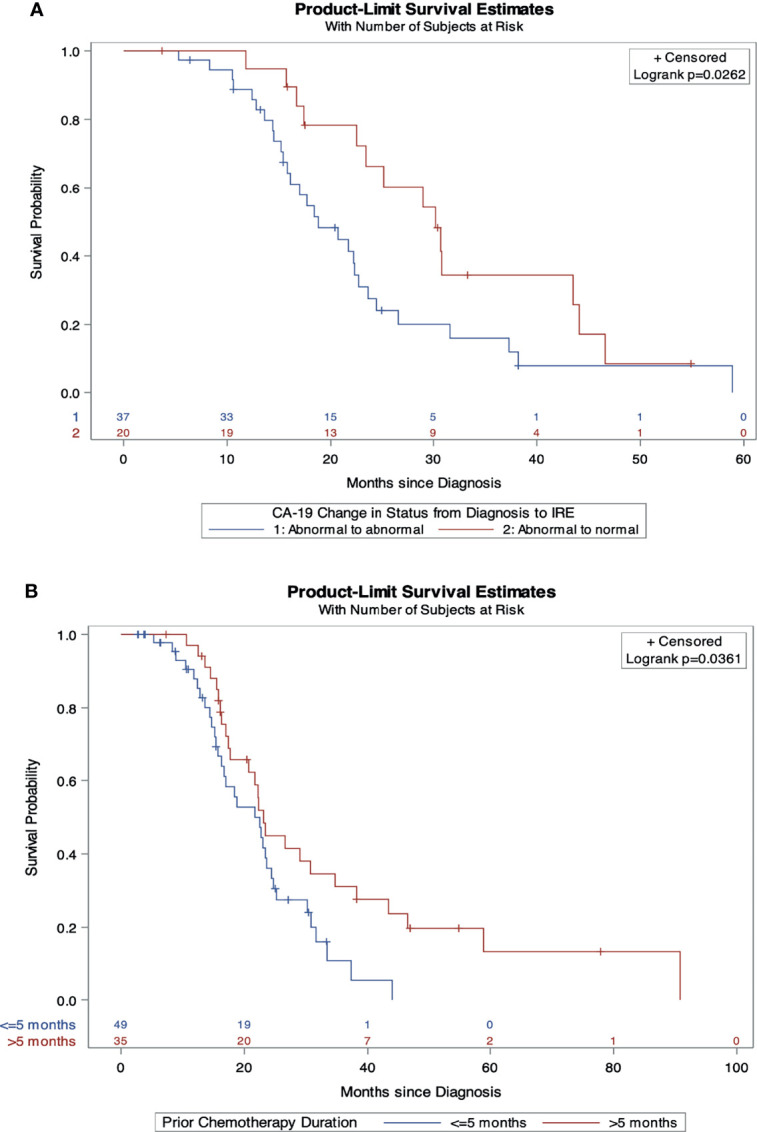
Independent predictors of overall survival from diagnosis. **(A)** Overall survival comparison by change in CA19-9 status from diagnosis to IRE. **(B)** Overall survival comparison by induction chemotherapy duration.

**Figure 2:**
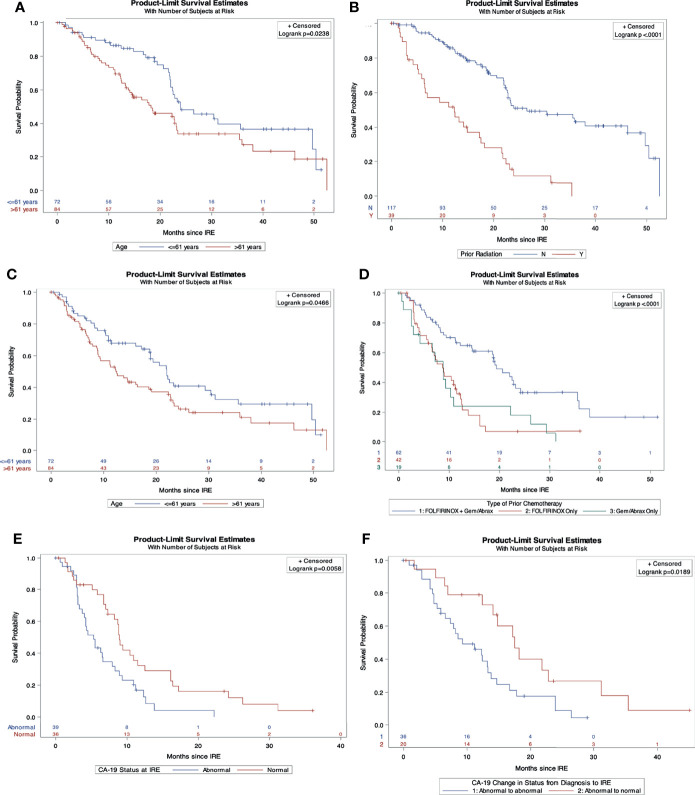
Independent predictors of overall and progression free survival from IRE. **(A)** Overall survival comparison by age. **(B)** Overall survival comparison by prior radiation history. **(C)** Progression free survival comparison by age. **(D)** Progression free survival comparison stratified by induction chemotherapy. **(E)** Overall survival comparison by CA19-9 status at IRE. **(F)** Progression free survival comparison of CA19-9 status from diagnosis to IRE.

On multivariable Cox regression analysis for independent predictive factors for survival, (**Table 5**) abnormal CA19-9 values at IRE (HR 2.16, 95% CI 1.1-4.2, p=0.02), and chemotherapy duration ≤ 5 months (HR 1.98, 95% CI 1.02-3.86, p=0.04) independently predicted a worse OS from diagnosis (**Figures 1 A, B**). However, age ≤ 61 years (HR 0.4, 95% CI, 0.21-0.78, p=0.008) and those without prior radiation history (HR 0.49, 95% CI 0.26-0.93, p=0.03) were independent predictors of improved in OS form IRE. Age ≤ 61 (HR 0.53, 95% CI 0.28-0.99, p=0.046) and FOLFIRINOX plus gemcitabine/abraxane induction chemotherapy, (HR 0.37, 95% CI 0.15-0.89, p=0.027) also predict improved PFS from IRE. Finally, abnormal CA19-9 at IRE was an independent predictor of both decreased OS (HR 2.46, 95% CI 1.28-4.72, p=0.007) and PFS (HR 2.19, 95% CI 1.143-4.2, p=0.018) (**Figures 2 A–F**) following IRE.

**Table 5:** Independent risk factors for survival in locally advanced pancreatic cancer patients.

	Adjusted HR (95% CI)	P value
**OS from diagnosis**		
CA19-9 change from diagnosis to IRE		
Abnormal to abnormal	2.159 (1.1-4.2)	**0.02**
Abnormal to normal	REF	
Induction chemotherapy duration		
*> 5 months*	1.98 (1.02-3.86)	**0.04**
* ≤ 5 months*	REF	
**OS from IRE**		
Age		
* ≤ 61*	0.4 (0.21-0.78)	**0.008**
* > 61*	REF	
CA19-9 at IRE		
* Abnormal*	2.46 (1.28-4.72)	**0.007**
* Normal*	REF	
Prior radiation		
* No*	0.49 (0.26-0.93)	**0.03**
* Yes*	REF	
**PFS from IRE**		
Age		
* ≤ 61*	0.53 (0.28-.99)	**0.046**
* > 61*	REF	
Induction chemotherapy		
* FOLFIRINOX +gemcitabine/abraxane*	0.37 (0.15-0.89)	**0.027**
* FOLFIRINOX only*	1.032 (0.468-2.275)	0.93
* Gemcitabine/abraxane*	REF	
CA19-9 at IRE		
Abnormal	2.192 (1.143-4.201)	**0.018**
Normal	REF	

OS, overall survival; HR, hazard ratio; CI, confidence interval; CA19-9, cancer antigen 19-9; IRE, irreversible electroporation; FOLFIRINOX, folinic acid, 5-fluorouracil, irinotecan, and oxaliplatin. Bold values represent statistical significance with p values less than 0.05.

## Discussion

The most important finding from this study is the identification of clinicopathologic characteristics that predict survival following open *in-situ* IRE for LAPC, which has not been previously established. Significant progress in oncologic management of stage III LAPC has occurred in the past decade (30). Total neoadjuvant chemotherapy in the setting of metastatic and locoregional pancreatic adenocarcinoma has improved survivals and allowed for more aggressive and consolidative operative interventions (5, 31–33). IRE in the setting of LAPC is one example and was first described in 2012, and has been proven safe near vital vessels and ductal structures due to its non-thermal mechanism of action (7). IRE has further proven to be an effective palliative surgical intervention with remarkable improvements in OS and PFS for those diagnosed with LAPC (8, 12, 34). Therefore, a better understanding of preoperative factors to assist in selecting patients to undergo IRE is now critical with the establishment of these key outcome measures.

The median PFS of 21.7 months and OS of 25.5 months in these 187 LAPC patients treated with open *in-situ* IRE, confirms that of previous reports and warranted this investigation into the selection process within this registry (8, 11, 12, 34–36). Earlier publications demonstrated variation in OS when utilizing IRE for LAPC (37–39). However, many factors may explain this underlying discrepancy. IRE has a demonstrable learning curve and over time with performance of more cases, allows for completion of complex ablations involving larger tumors and those with a high degree of vascular involvement (40). Differences in tumor biology, heterogeneity of NAC regimens, patient selection, and approach or technique (open vs laparoscopic vs percutaneous) technique may also attribute. Lack of energy delivery standardization also greatly limits the reproducibility of results. Inadequate energy delivery to the tumor leading to incomplete ablations or reversible electroporation can actually increase tumor growth (26, 41). All participating institutions within this study adhered to the recommended AHPBA IRE technical recommendations. We encourage, all centers performing IRE to follow these guidelines with respect to individual patient and tumor related factors in a concerted effort improve safety, reproducibility of results, and facilitate ongoing research (21). In addition, patients should not be excluded from IRE treatment if they do not meet all of these criteria but need to be informed as to their risk and long term outcomes. This data supports the appropriate use of IRE in LAPC and emphasizes that better patient selection can lead to survivals of close to 24 months.

Here we observed age > 61, > 2 comorbidities, and those with diabetes to negatively impact OS and PFS following IRE. These findings are consistent with reports seen in other pancreatic adenocarcinoma patient populations (4, 42–44). Age less than 61 at electroporation also independently predicted prolonged PFS and OS following IRE in this cohort. It is well known that the incidence of PDAC positively correlates with age (45). Several population-based studies have reported on poorer prognoses in older PDAC patients (46, 47). Wang et al. recently reported on 126,066 patients from the National Cancer Institute’s Surveillance, Epidemiology, and the End Results data base. Risk for mortality was double for those aged 40-80 years compared to PDAC patients less than 40 years (42). These findings are expected as PDAC is primarily a cancer of older age and physiologic reserve decreases over time. Elderly patients are also more likely to have increased frailty scores, which is a known independent predictor of mortality following pancreaticoduodenectomy (48–50). It should be noted that 41% of this cohort had performance statuses of 100%, which demonstrates our selection bias toward optimal function prior to operative intervention. The importance of performance status (PS) as a prognosticator in all stages of PDAC cannot be overstated (51). Every effort should be taken to optimize PS for LAPC patients presenting for IRE as nearly every patient will have undergone extensive induction chemotherapy leaving them malnourished and immune compromised. Prescribing preoperative nutritional supplements is one way providers can positively influence post-IRE outcomes (52).

In regard to comorbidities, many studies have established risk for the development of PDAC in the setting of diabetes mellitus (DM) (53). Additionally, our finding of DM negatively impacting OS (13.3 mo. vs. 23.1 mo., p<0.003) and PFS (20.6 mo. vs. 7.2 mo., p<0.0003) following IRE is in agreement with current knowledge (54–57). The effect of DM on survival has been demonstrated in both the short- and long-term settings. In 2015 Yuan et al., demonstrated significantly decreased OS in PDAC patients diagnosed with long term (>4 years) DM (58). Chu et al. also reported recent onset DM as an independent predictor of post resection survival (43). DM has also been significantly associated with increased tumor sizes and increased risk of death following pancreatectomy and adjuvant chemotherapy (59). Collectively, these data suggest at this time patients with DM are poor candidates to receive IRE for LAPC and warrants further investigations into treatment options for such an at risk population.

Anatomic tumor characteristics are also important to consider when evaluating patients for IRE therapy. Survival among LAPC patients following NAC and resection has been integrally tied to tumor size. Gemenetiz et al. found significantly prolonged OS in resected patients with smaller tumor sizes (33). Smaller tumors were also independently predictive of survival in an ancillary study of the LAP 07 trial (60). In agreement with these assessments, we have also found smaller tumor size (<3.6cm) to be predictive of PFS and OS following IRE on univariate analyses. In addition, patients with vascular involvement ≤180 degrees of their affected structure are more likely to have significantly longer OS from their initial diagnoses. Thus, it appears IRE may have its greatest impact on tumors of smaller size with less circumferential vessel involvement. Surgeons and interventionalists performing IRE should be cognizant of these tumor qualities and discuss in such cases in a multidisciplinary setting prior to proceeding.

This data again highlights the important prognostic value of CA19-9 in LAPC and adds value to its ability to be used as a treatment biomarker. Serum measurement of CA19-9 as a surrogate for clinical outcomes in pancreatic cancer is well established (61–63). However, only recently have we begun to accept CA19-9 monitoring to guide multimodality therapy. Following NAC, normalization of CA19-9 has been reported to be a strong prognostic marker for long term survival (64–66). Here, patients who achieved normative CA19-9 levels at the time of IRE were able to achieve nearly double the survivals of those with abnormal values. This reiterates that CA19-9 should be used to guide multimodality therapy and suggests those with good response may be better candidates for further consolidative therapy.

The effectiveness of IRE as a consolidative therapy in conjunction with systemic chemotherapy and/or chemoradiation is becoming better understood. However, current NCCN guidelines continue to be heterogeneous in chemotherapeutic recommendations for stage III LAPC (67). This array of treatment options may allow for more tolerable treatment to be prescribed yet with limited efficacy that may confound key outcomes. The success of FOLFIRINOX in treatment of LAPC or borderline resectable disease calls for more standardization of preoperative treatment (5, 68, 69). Our finding that FOLFIRINOX and gemcitabine/abraxane therapy to be an independent predictor of PFS following IRE supports the focused use of these modern induction agents in LAPC prior to IRE. We have also seen those who receive induction chemotherapy for > 5 months have significantly improved OS from diagnosis. This time allows for a thorough assessment of patient disease and for optimal response evaluation. Furthermore, the delivery of electroporation with these chemotherapeutics (i.e. electrochemotherapy) improves the delivery of such agents to a complex tumor microenvironment (TME) with synergistic anti-tumor activity (70, 71). With respect to radiation, use of radiotherapy in the setting of LAPC has historically been controversial, however current NCCN guidelines recommend its use. Though, data surrounding its utility in LAPC is fraught with conflicting evidence (72–75). While there are many studies investigating radiation in LAPC, none have focused on patients receiving IRE. Here we have found radiation to be a negative predictor of OS following IRE. Activating tumor molecular phenotypic changes as a result of chemoradiation such as persistence of stellate cells, cleavage of caspases, or protein kinase causing tumor activation has been described (76). However, at this time as an explanation of these findings would be speculative at best. Certainly, more research is needed to investigate the impact radiation has in the setting of IRE and the interplay of their mechanisms of action within the TME.

**Figure 3:**
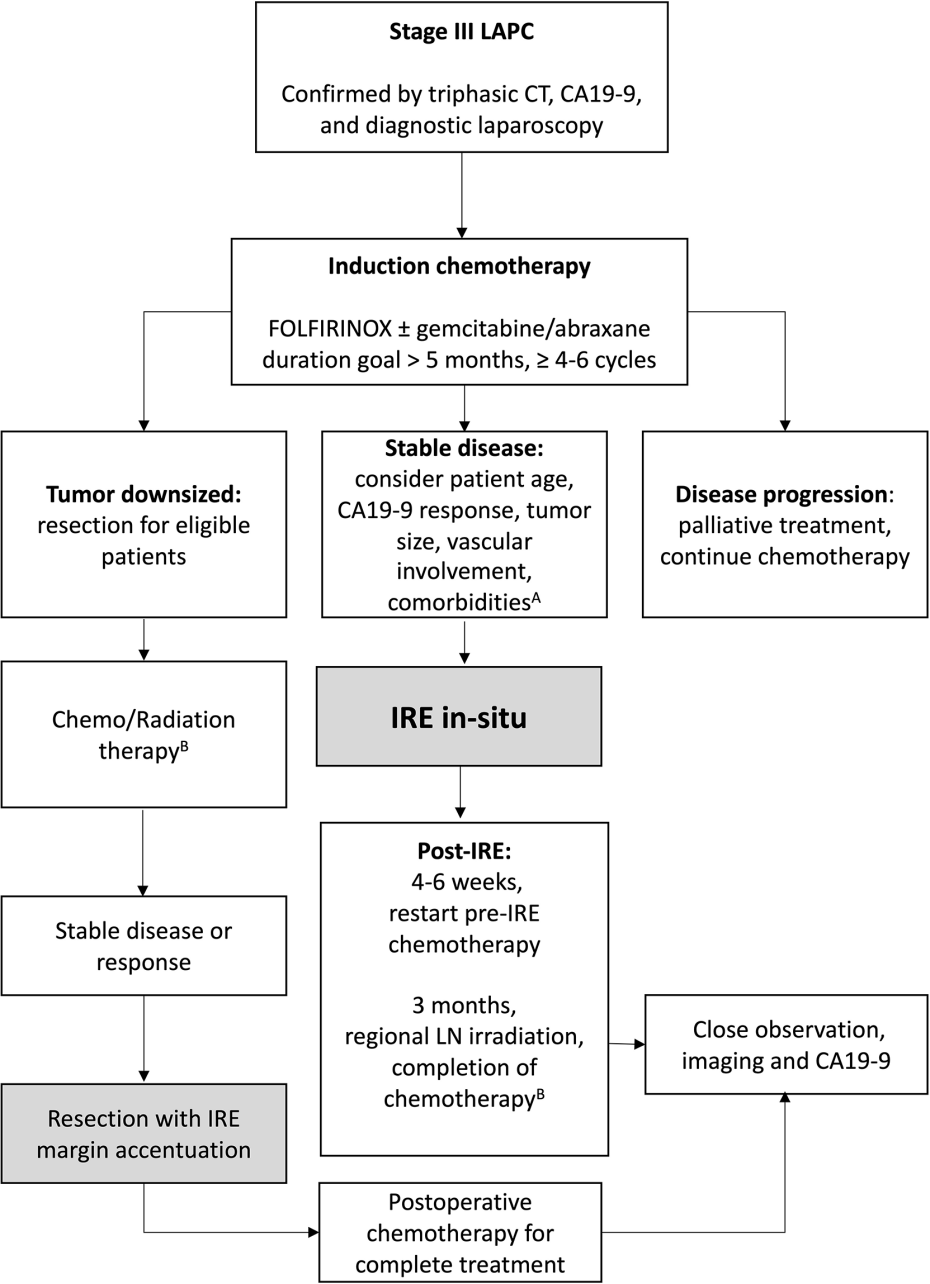
Patient selection algorithm. A) Optimal patient selection criteria: Age ≤ 61, normalized CA19-9 at IRE, tumor size < 3.6cm, ≤ 180° vascular involvement, less than 2 co-morbidities, and those without diabetes mellitus. B) Can continue FOLFIRINOX (per PRODIGE trial). Chemotherapy goal 12 cycles of FOLFIRINOX, 18 total doses gemcitabine/abraxane.

IRE has been described as a last resort, with some practitioners referring for intervention once patients have become otherwise unresponsive to systemic chemotherapy. For example, Piella et al. reported on 10 patients with unresponsive LAPC who underwent IRE and found median OS of 7.5 months (38). In light of the present findings, we want to strongly encourage the medical oncology community to refer for IRE in patients whose biology of disease and clinical characteristics would positively favor their response to IRE. To that end, we have recommended a specific treatment algorithm that optimizes all three active treatments in the management of LAPC (**Figure 3**). We believe it is critical to understand that potential substantial benefit can be obtained when all of these favorable prognostic factors are achieved, but we also want to emphasize that improvements in overall outcomes can be achieved even in patients who may not meet all of these prognostic factors and represents a guide for patient selection and for future management and comparisons.

The present study should be interpreted with respect to the following limitations. First, lack of randomization in this prospective cohort limits ability to determine the degree of survival benefit patients received from induction chemotherapy and IRE. Conventional imaging modalities to detect immune relevant responses are lacking and therefore determination of recurrence or progressive disease based on current RECSIST guidelines are likely underestimated. Additionally, there was a degree of post IRE imaging variability between participating institutions. As previously reported, this cohort is prone to selection bias. The participating centers have carefully selected patients who do not progress on systemic chemotherapy, with enhanced performance statuses, and limited co-morbidities to receive IRE. These limitations notwithstanding, this study is the most comprehensive and only prospective multi-institution evaluation for prognosticators of survival in the setting of LAPC treated with open *in-situ* IRE to date. Until now, the optimal patient characteristics highlighting improved OS and PFS after IRE for LAPC were not elucidated.

## Conclusions

This prospective cohort evaluation of stage III LAPC patients treated with open IRE demonstrates prominent factors predictive of PFS and OS that should be used to aide in selection or referral for patients to receive open technique IRE. These results demonstrate that prolonged survival beyond historical controls can be achieved by IRE of LAPC in appropriately selected patients. This study further supports the design of randomized multi-center clinical trials investigating the efficacy of IRE, which are now actively recruiting participants (NCT03899636, NCT03899649).

## Data Availability Statement

The original contributions presented in the study are included in the article/**Supplementary Material**. Further inquiries can be directed to the corresponding author.

## Ethics Statement

The studies involving human participants were reviewed and approved by U of L IRB. The patients/participants provided their written informed consent to participate in this study.

## Author Contributions

MRW, KW, EK, MJW, JC, RW, and RMII contributed to study design and manuscript revisions. MRW, KW, and RMII contributed to study design, drafting of the manuscript, and manuscript revisions. EK, MJW, JC, RW, and RMII contributed to study design, data acquisition, and manuscript revisions. RM contributed to study design, data acquisition, analysis, drafting of the manuscript, and manuscript revisions. All authors contributed to the article and approved the submitted version.

## Conflict of Interest

RM II, MD, PhD, FACS is an educational consultant for AngioDynamics, Inc.

The remaining authors declare that the research was conducted in the absence of any commercial or financial relationships that could be construed as a potential conflict of interest.

## Publisher’s Note

All claims expressed in this article are solely those of the authors and do not necessarily represent those of their affiliated organizations, or those of the publisher, the editors and the reviewers. Any product that may be evaluated in this article, or claim that may be made by its manufacturer, is not guaranteed or endorsed by the publisher.

## Supplementary Material

Supplementary Figure 1 Progression free survival of patients who underwent Pancreatectomy with IRE for margin accentuation versus patients who underwent IRE alone (*In-Situ*)

Supplementary Table 1 Observed adverse events.
