# Supplementary figures and images for: Optimizing Patient Selection for Irreversible Electroporation of Locally Advanced Pancreatic Cancer: Analyses of Survival
Source: Front Oncol. 2022 Jan 13;11:817220. doi: 10.3389/fonc.2021.817220 (PMC8793779; doi:10.3389/fonc.2021.817220)

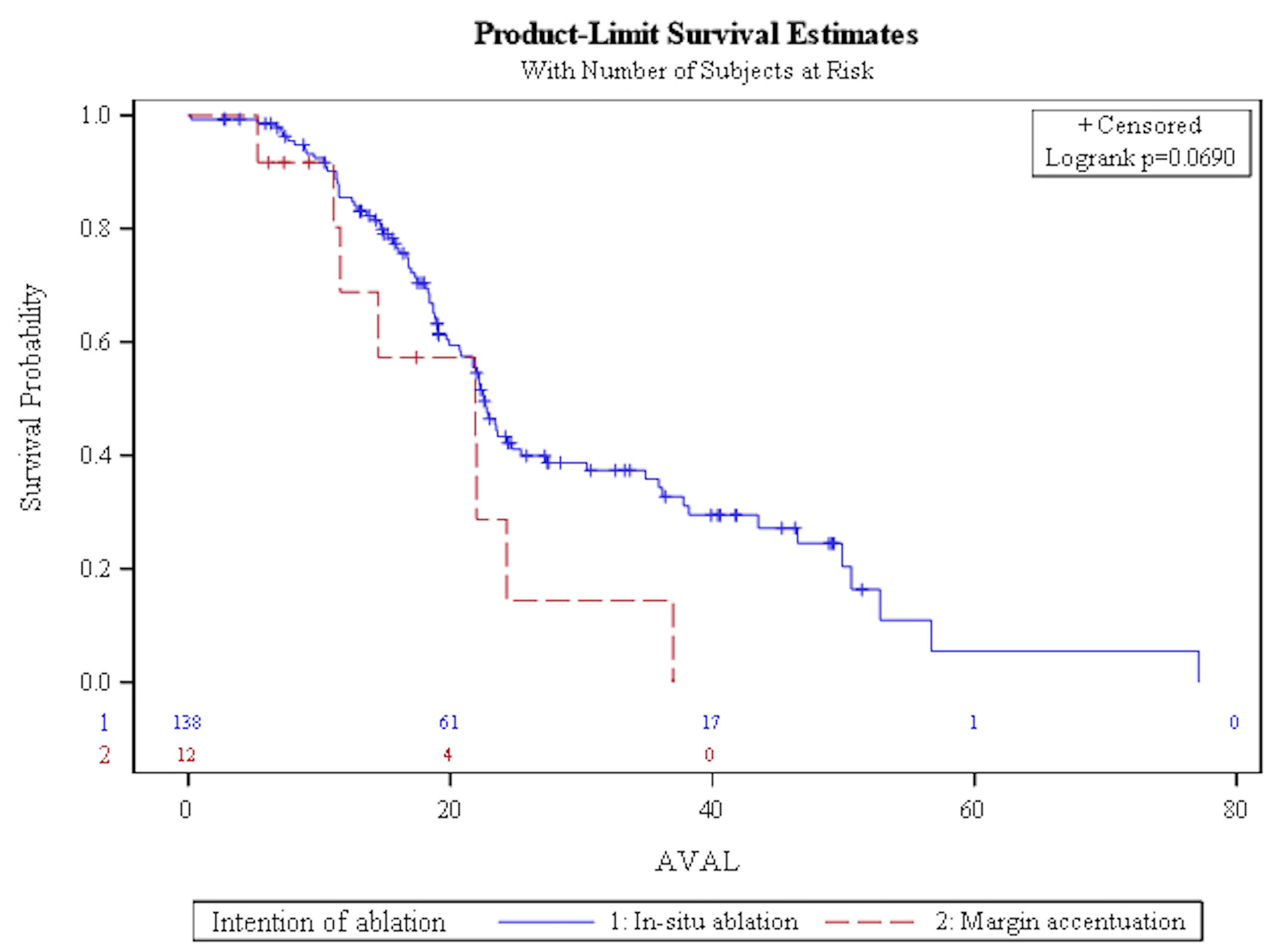

Supplement: Supplementary Figure 1 — Progression free survival of patients who underwent Pancreatectomy with IRE for margin accentuation versus patients who underwent IRE alone (In-Situ) [file Image_1.jpeg]
