# Supplementary material for: Optimizing Patient Selection for Irreversible Electroporation of Locally Advanced Pancreatic Cancer: Analyses of Survival
Source: Front Oncol. 2022 Jan 13;11:817220. doi: 10.3389/fonc.2021.817220 (PMC8793779; doi:10.3389/fonc.2021.817220)
Supplement: Supplementary Table 1 — Observed adverse events. [file Table_1.docx]

**Supplementary table 1: Observed adverse events**

| **CTCAE Term, n (%)** | **Grade 3** | **Grade 4** | **Grade 5** |
| --- | --- | --- | --- |
| AKI | 2 (1) | 0 | 0 |
| Liver abscess | 2 (1) | 0 | 0 |
| Anxiety | 1 (0.5) | 0 | 0 |
| Ascites | 5 (3) | 0 | 0 |
| Bacteremia | 1 (0.5) | 1 (0.5) | 0 |
| Bile duct obstruction | 1 (0.5) | 0 | 0 |
| Bile leak | 1 (0.5) | 0 | 0 |
| Clostridium difficile colitis | 1 (0.5) | 0 | 0 |
| Cardiac arrest | 0 | 0 | 1 (0.5) |
| T1 paralysis | 0 | 0 | 1 (0.5) |
| DVT | 1 (0.5) | 0 | 0 |
| Duodenal perforation | 0 | 1 (0.5) | 0 |
| Empyema | 1 (0.5) | 0 | 0 |
| Fungal peritonitis | 1 (0.5) | 0 | 0 |
| GI bleed | 5 (3) | 1 (0.5) | 1 (0.5) |
| Gastritis-colitis | 2 (1) | 0 | 0 |
| Hypoxia | 1 (0.5) | 0 | 0 |
| Malnutrition | 0 | 0 | 1 (0.5) |
| Pancreatic pseudoaneurysm | 1 (0.5) | 0 | 0 |
| Pancreatitis | 1 (0.5) | 0 | 0 |
| Peripancreatic fluid collection | 1 (0.5) | 0 | 0 |
| Pleural effusion | 1 (0.5) | 0 | 0 |
| Pneumatosis | 1 (0.5) | 0 | 0 |
| Postoperative bleeding | 1 (0.5) | 0 | 0 |
| GDA pseudoaneurysm | 1 (0.5) | 0 | 0 |
| Pseudomembranous colitis | 1 (0.5) | 0 | 0 |
| Renal failure | 0 | 0 | 1 (0.5) |
| Sepsis | 0 | 1 (0.5) | 1 (0.5) |
| Delayed gastric emptying | 1 (0.5) | 0 | 0 |
| Splenic aneurysm | 0 | 1 (0.5) | 0 |
| Kidney stones | 1 (0.5) | 0 | 0 |
| PNA | 1 (0.5) | 0 | 0 |

CTCAE: Common terminology criteria for adverse events; AKI: acute kidney injury; DVT: deep venous thrombosis; GI: gastrointestinal; GDA: gastroduodenal artery; PNA: pneumonia
